# Differences in Response and Surgical Management with Neoadjuvant Chemotherapy in Invasive Lobular Versus Ductal Breast Cancer

**DOI:** 10.1245/s10434-015-4603-3

**Published:** 2015-05-16

**Authors:** W. Truin, G. Vugts, R. M. H. Roumen, A. J. G. Maaskant-Braat, G. A. P. Nieuwenhuijzen, M. van der Heiden-van der Loo, V. C. G. Tjan-Heijnen, A. C. Voogd

**Affiliations:** Department of Surgery, Máxima Medical Centre, Veldhoven, The Netherlands; Department of Surgery, Catharina Hospital, Eindhoven, The Netherlands; Department of Research, Netherlands Comprehensive Cancer Organization IKNL, Utrecht, The Netherlands; Department of Medical Oncology, GROW–School for Oncology and Developmental Biology, Maastricht University Medical Centre, Maastricht, The Netherlands; Department of Epidemiology, Maastricht University Medical Centre, Maastricht, The Netherlands

## Abstract

**Background:**

This study was conducted to determine the impact of neoadjuvant chemotherapy (NAC) on the likelihood of breast-conserving surgery (BCS) performed for patients with invasive lobular breast carcinoma (ILC) and invasive ductal carcinoma (IDC).

**Methods:**

Female patients with a diagnosis of ILC or IDC in The Netherlands between July 2008 and December 2012 were identified through the population-based Netherlands Cancer Registry.

**Results:**

A total of 466 ILC patients received NAC compared with 3622 IDC patients. Downstaging by NAC was seen in 49.7 % of the patients with ILC and in 69.6 % of the patients with IDC, and a pathologic complete response (pCR) was observed in 4.9 and 20.2 % of these patients, respectively (*P* < 0.0001). Breast-conserving surgery was performed for 24.4 % of the patients with ILC receiving NAC versus 39.4 % of the patients with IDC. In the ILC group, 8.2 % of the patients needed surgical reinterventions after BCS due to tumor-positive resection margins compared with 3.4 % of the patients with IDC (*P* < 0.0001). Lobular histology was independently associated with a higher mastectomy rate (odds ratio 1.91; 95 % confidence interval 1.49–2.44). Among the patients with clinical T2 and T3 disease, BCS was achieved more often when NAC was administered in ILC as well as IDC.

**Conclusion:**

The patients with ILC receiving NAC were less likely to experience a pCR and less likely to undergo BCS than the patients with IDC. With regard to BCS, the impact of NAC for ILC patients was lower than for patients receiving surgery without NAC. However, despite the high number to treating in order to achieve BCS, a small subset of ILC patients, especially cT2 and cT3 patients, still may benefit from NAC.

Invasive lobular carcinoma (ILC) is the second most common type of breast cancer, constituting 5–15 % of all histologic types of breast cancer.^1^ Due to its specific clinical, biologic, and prognostic features, ILC often is considered to be a distinct clinical entity different from invasive ductal carcinoma (IDC). Patients with ILC present with significantly larger tumors at the time of diagnosis and more often show multifocal or multicentric disease.^2^,^3^ The diffuse infiltrative growth pattern of ILC poses a difficulty in determining the extent of the tumor.^4^,^5^ As a result of these characteristics, higher rates of positive surgical resection margins are observed in the primary surgical procedure in ILC compared with IDC.^6^ This results in higher rates of re-resection and completion mastectomy for patients with lobular histology.^7^,^8^

Neoadjuvant chemotherapy (NAC) is increasingly used in the treatment of patients with breast cancer. The NAC approach has several objectives, including downsizing of irresectable locally advanced breast cancer into operable disease. Furthermore, it allows in vivo monitoring of the tumor’s chemosensitivity and also gives the opportunity for downstaging of disease in the axilla, obviating the need for axillary treatment in some patients. However, from a surgical point of view, the most important objective of NAC is to increase the possibility that breast-conserving surgery (BCS) can be performed.^9^

Invasive lobular carcinoma is known to be less responsive to NAC. The reported proportions of ILC patients with a pathologic complete response (pCR) range from 1 to 3 % compared with 9 to 15 % of IDC patients.^10^,^11^ Furthermore, in a small study including patients with ILC, NAC did not appear to increase the likelihood of breast conservation.^12^ Also, when treated with NAC, patients with ILC have been more likely to have positive surgical margins than patients with IDC. In a study by Soucy et al.^13^ 43 % of the patients with ILC had positive margins compared with 16 % of those with IDC (*P* = 0.002).^14^

In this study, we determined the surgical benefit of NAC for patients with ILC by comparing the use of BCS and the performance of re-resections after NAC between ILC and IDC patients in a nationwide Dutch prospective cohort.

## Methods

### Patients

All female patients with ILC or IDC diagnosed between July 2008 and December 2012 (*n* = 53,929) were selected from the population-based Netherlands Cancer Registry (NCR). The registry records data on all patients with a new diagnosis of in situ and invasive tumors in the Netherlands. Trained registry managers prospectively collect data from medical records after notification, which are mainly obtained from the automated pathology archive (PALGA). Other sources used are the National Registry of Hospital Discharge Diagnoses and the databases of the radiotherapy departments. Specially trained registration clerks collect data about patient, tumor, and treatment characteristics from patient hospital files. Due to thorough registrar training, computerized consistency checks, and regular national quality checks, the quality of the data is considered high.^15^

For this study, data concerning patient and tumor characteristics and use of NAC were derived from the Netherlands Cancer Registry. Patients with pure invasive ductal or lobular histology were included, whereas patients with mixed type and other carcinomas were excluded from further analyses. Also, patients who presented with primary metastatic breast cancer were excluded. Primary tumor stage was based on the tumor-node-metastasis (TNM) Classification of Malignant Tumors by the International Union Against Cancer (UICC), 6th edition.^16^

### Statistical Analyses

Trends in the administration of NAC for ILC patients versus IDC patients were recorded. In the cohort of patients treated with NAC, we analyzed pCR in the breast for ILC versus IDC. Besides pCR, we also evaluated downstaging after NAC. Because the exact clinical and pathologic tumor diameters were not available from the NCR, downstaging was defined as a ypT status (pT status after NAC and pathological analysis of the surgical specimen), which was lower than the clinical T status (cT) before NAC.

Finally, the rate of BCS was compared between patients who received NAC and those who underwent primary surgical treatment, stratified for cT status. Data were analyzed using Statistical Analysis System (SAS) version 9.3 (SAS Institute Inc., Cary, NC). To compare the proportion of patients receiving NAC and the stage distribution after NAC between patients with ILC and IDC, the *χ*^2^ test was used. Means of continuous variables were compared using the independent-sampled Student’s *t* test. A *P* value lower than 0.05 was considered statistically significant. The percentage of BCS and the percentage of re-excisions between ILC and IDC patients also were compared using the *χ*^2^ test. A logistic regression analysis was performed to determine independent predictors for BCS after NAC, including histologic tumor type, receptor status, clinical tumor size, and age.

## Results

### Population

Between July 2008 and December 2012, ILC or IDC was diagnosed for 53,929 female patients. Of the 6401 patients with ILC, 466 (7.3 %) received NAC versus 3622 (8.1 %) of the 44,597 patients with IDC (*P* = 0.02). At diagnosis, the patients with ILC were older (median age 52 years) than the patients with IDC (median age 49 years) (*P* < 0.0001) (Table 1). The patients with ILC were more likely to have a positive estrogen receptor (ER) and progesterone receptor (PR) status, whereas the patients with IDC more often presented with HER2-positive tumors (Table 1). Among the patients with ILC, the use of NAC increased from 6.0 % in 2008 to 8.6 % in 2011, but in 2012, it decreased to 7.8 %. Among the patients with IDC, the use of NAC increased from 6.7 % in 2008 to 9.8 % in 2012.

**Table 1:** Comparison of clinical and pathologic tumor characteristics between patients receiving neoadjuvant chemotherapy (NAC) and patients receiving primary surgery

	ILC	IDC	*P* value
Median age: years (range)	52.0 (47.0–60.0)	49.0 (43.0–58.0)	<0.0001
Patients receiving NAC	466	3622	
Clinical tumor size			<0.0001
T1	35 (7.5 %)	405 (11.2 %)	
T2	193 (41.4 %)	1896 (52.3 %)	
T3	183 (39.3 %)	728 (20.1 %)	
T4	54 (11.6 %)	586 (15.7 %)	
Unknown	1 (0.2 %)	7 (0.2 %)	
Pathologic tumor size			<0.0001
T0	23 (4.9 %)	739 (20.4 %)	
T1	128 (27.5 %)	1444 (39.9 %)	
T2	162 (34.8 %)	773 (21.3 %)	
T3	96 (20.6 %)	188 (5.2 %)	
T4	8 (1.7 %)	82 (2.3 %)	
Unknown	49 (10.5 %)	396 (10.9 %)	
Receptor status			
ER+	434 (93.1 %)	2400 (66.3 %)	<0.0001
PR+	343 (73.6 %)	1818 (50.2 %)	<0.0001
HER2+	35 (7.5 %)	973 (26.7 %)	<0.0001
Patients not receiving NAC	5935	40,975	
Clinical tumor size			<0.0001
Tis	39 (0.7 %)	804 (2.0 %)	
T1	3253 (54.8 %)	26,582 (64.9 %)	
T2	1921 (32.4 %)	10,909 (26.6 %)	
T3	409 (6.9 %)	561 (1.4 %)	
T4	47 (0.8 %)	308 (0.8 %)	
Unknown	266 (4.5 %)	1721 (4.2 %)	
Pathologic tumor size			<0.0001
T0	3 (0.1 %)	14 (0.0 %)	
T1	3065 (51.6 %)	27,848 (68.0 %)	
T2	2245 (37.8 %)	12,053 (29.4 %)	
T3	560 (9.4 %)	622 (1.5 %)	
T4	29 (0.5 %)	233 (0.6 %)	
Unknown	33 (0.6 %)	205 (0.5 %)	
Receptor status			
ER+	5702 (96.1 %)	33,470 (81.7 %)	<0.0001
PR+	4362 (73.5 %)	26,642 (65.0 %)	<0.0001
HER2+	236 (4.0 %)	5662 (13.8 %)	<0.0001
Total no. of patients	6401	44,597	

*ILC* invasive lobular carcinoma, *IDC* invasive ductal carcinoma, *ER* estrogen receptor, *PR* progesterone receptor, *HER* human epidermal growth factor receptor

### Response After NAC

Of all the ILC patients treated with NAC, 218 (46.8 %) experienced downstaging of their tumor compared with 2355 of the IDC patients (65 %) (*P* < 0.0001). Among the patients with ILC, downstaging occurred for 11.4 % of those with cT1 tumors, 36.3 % of those with cT2 tumors, 57.4 % of those with cT3 tumors, and 72.2 % of those with cT4 tumors. Among the patients with IDC, these figures were 27.4 % for cT1 tumors, 66.3 % for cT2 tumors, 73.9 % for cT3 tumors, and 76.6 %, for cT4 tumors. The overall pCR rate for the patients with ILC was 4.9 % compared with 20.2 % for the patients with IDC (*P* < 0.0001). Among the patients with ILC and those with IDC, downstaging of the primary tumor after NAC was more likely to be achieved in those presenting with larger tumors. The ILC patients who experienced downstaging of their tumor after NAC less frequently had ER-positive tumors (88.5 vs. 97.2 %; *P* = 0.0002) and PR-positive tumors (67.9 vs. 78.6 %; *P* = 0.0087) than the ILC patients in whom downstaging did not occur.

No difference was observed in the proportion of ILC patients with HER2-positive disease between those with and those without downstaging. Among the patients with IDC, those with downstaging after NAC also were less likely to have ER-positive disease (62.8 vs. 72.7 %; *P* < 0.0001) or PR-positive disease (47.5 vs. 55.2 %; *P* < 0.0001) and more likely to have HER2-positive disease (28.2 vs. 24.4 %; *P* = 0.0137).

### Surgery After NAC

Among the ILC patients treated with NAC, BCS was the primary surgical procedure in 24.2 % (*n* = 113) and mastectomy in 75.8 % (*n* = 353; Table 2). Among the patients with ILC, 38 (8.2 %) needed a reexcision of margins to obtain local control. Ultimately, mastectomy was performed for 82.2 % of all the patients with ILC (*n* = 383). Among the patients with IDC, BCS was the primary surgical intervention for 39.4 % (*n* = 1426) and mastectomy for 60.6 % (*n* = 2196; Table 2). Of the patients with IDC, 3.4 % underwent a re-excision of margins to obtain local control. Ultimately, 62.5 % of the patients with IDC underwent mastectomy, which was significantly lower than the 82.2 % for the patients with ILC (*P* < 0.0001).

**Table 2:** Type of surgery after neoadjuvant chemotherapy (NAC)

	ILC (*n* = 466)		IDC (*n* = 3622)		*P* value
	Mastectomy	BCS	Mastectomy	BCS	
Initial surgery					
Clinical tumor size					
T1	18 (51.4 %)	17 (48.6 %)	208 (51.4 %)	197 (48.6 %)	0.9936
T2	119 (61.7 %)	74 (38.3 %)	840 (44.3 %)	1056 (55.7 %)	<0.0001
T3	163 (89.1 %)	20 (10.9 %)	590 (81.0 %)	138 (19.0 %)	0.0104
T4	52 (96.3 %)	2 (3.7 %)	551 (94.0 %)	35 (6.0 %)	0.4942
Unknown	1	0	7	0	
Total	353 (75.8 %)	113 (24.4 %)	2196 (60.6 %)	1426 (39.4 %)	<0.0001
Surgical reinterventions					<0.0001
BCS	8 (1.7 %)		56 (1.5 %)		
Mastectomy	30 (6.4 %)		67 (1.8 %)		
Definitive surgery					<0.0001
BCS	83 (17.8 %)		1359 (37.5 %)		
Mastectomy	383 (82.2 %)		2263 (62.5 %)		

*ILC* invasive lobular carcinoma, *IDC* invasive ductal carcinoma, *BCS* breast-conserving surgery

The ILC patients with cT2 and cT3 tumors more often underwent a primary mastectomy than those with IDC. These differences were not apparent in the patients with cT1 and cT4 tumors (Table 2).

A logistic regression analysis showed that histology was an independent predictor for the use of BCS (Table 3). The patients with IDC were more likely to undergo BCS than the patients with ILC [odds ratio (OR) 1.91; 95 % confidence interval (CI) 1.49–2.44]. This analysis also showed that only a cT2 tumor was associated with a greater chance of BCS after NAC. No improvement in BCS rates for cT3 and T4 tumors occurred after NAC in either the ILC or IDC patients.

**Table 3:** Multivariate analysis of factors associated with the chance of breast-conserving surgery (BCS) after neoadjuvant chemotherapy (NAC)

	OR	95 % CI
Histology		
ILC	1	
IDC	1.91	1.49–2.44
Clinical tumor size		
T1	1	
T2	1.25	1.01–1.54
T3	0.23	0.18–0.30
T4	0.07	0.04–0.10
Age (years)		
<50	1	
50–70	1.35	1.17–1.56
>70	0.59	0.34–1.02
Estrogen receptor status		
Negative	1	
Positive	0.84	0.68–1.04
Progesterone receptor status		
Negative	1	
Positive	1.11	0.92–1.35
HER2 receptor status		
Negative	1	
Positive	0.95	0.81–1.13

*OR* odds ratio, *CI* confidence interval, *ILC* invasive lobular carcinoma, *IDC* invasive ductal carcinoma, *HER* human epidermal growth factor receptor

### Effect of NAC on Breast Conservation

To determine the effect of NAC on the use of breast conservation, we compared the patients who underwent NAC with those who underwent primary surgery without NAC. The patients who underwent NAC presented with significantly larger tumors than the patients who underwent primary surgery without NAC. In the group of ILC patients treated with NAC, 51 % presented with cT3 or cT4 breast cancer. For the IDC patients, this was 36 %. Of all the patients with cT3 or cT4 breast cancer, the proportion that underwent primary surgery without NAC was 7.7 % for those with ILC and 2 % for those with IDC (Table 4). Among the patients with ILC and cT2 tumors, 38.3 % underwent BCS when NAC was used compared with 29.6 % when NAC was not administered (*P* < 0.0001). Of the patients with ILC and cT3 tumors, 10.9 % of those who had NAC underwent BCS compared with 4.4 % of those undergoing surgery without NAC (*P* < 0.0001). Among the patients with IDC, BCS was achieved in 55.7 % of those with cT2 tumors receiving NAC compared with 44.0 % of those who had cT2 tumors without NAC (*P* < 0.0001). In the IDC patients with cT3 tumors, BCS was achieved in 19.0 % of those with NAC compared with 5.1 % in those without NAC (*P* < 0.0001). When BCS is set as a primary goal of NAC, in lobular histology, the number needed to treat (NNT) is 11.5 for cT2 tumors and 15.4 for cT3 tumors. In patients with IDC, the NNT to achieve BCS after NAC is 8.5 for cT2 tumors and 7.2 for cT3 tumors.

**Table 4:** Surgical treatment in patients after neoadjuvant chemotherapy (NAC) versus primary surgery for in invasive lobular carcinoma (ILC) versus invasive ductal carcinoma (IDC) patients

	Patients receiving NAC		Patients not receiving NAC		*P* value
	Mastectomy	BCS	Mastectomy	BCS	
ILC	466		5935		
Clinical tumor size					
T1	18 (51.4)	17 (48.6)	1191 (36.6)	2062 (63.4)	<0.0001
T2	119 (61.7)	74 (38.3)	1352 (70.4)	569 (29.6)	<0.0001
T3	163 (89.1)	20 (10.9)	391 (95.6)	18 (4.4)	<0.0001
T4	52 (96.3)	2 (3.7)	40 (85.1)	7 (14.9)	0.0042
All ILC patients	353 (75.6)	113 (24.4)	3119 (52.5)	2816 (47.5)	<0.0001

IDC	3622		40,975		
Clinical tumor size					
T1	208 (51.4)	197 (48.6)	7133 (26.8)	19,449 (73.2)	<0.0001
T2	840 (44.3)	1056 (55.7)	6106 (56.0)	4803 (44.0)	<0.0001
T3	590 (81.0)	138 (19.0)	528 (94.9)	33 (5.1)	<0.0001
T4	551 (94.0)	35 (6.0)	272 (89.9)	31 (10.1)	0.0262
All IDC patients	2196 (60.6)	1426 (39.4)	15,174 (37.0)	25,801 (63.0)	<0.0001

Clinical tumor size was not known for 1 ILC and 7 IDC patients receiving NAC or for 266 ILC and 1721 IDC patients undergoing surgery without NAC

*BCS* breast-conserving surgery

## Discussion

The impact of breast cancer histology on the use of breast conservation and on the risk of positive resection margins and reinterventions is well established. Since the introduction of NAC, BCS can be achieved more often for patients with initially large tumors. However, the surgical benefits of NAC for patients with breast cancer of lobular histology compared with ductal histology remain unclear.

In this large, population-based cohort study of 466 ILC patients and 3622 IDC patients treated with NAC, we confirmed that lobular histology is associated with a smaller probability of response and pCR after NAC. Furthermore, compared with IDC, fewer ILC patients were treated with BCS after NAC. When treatment started with BCS, the patients with ILC were more likely to undergo a margin re-excision than the patients with IDC. Both lobular histology and poor response to NAC were independently associated with a higher mastectomy rate. However, a significant beneficial effect of NAC on BCS for patients with cT2 and cT3 ILC was observed, although this effect was smaller than the effect in IDC.

A reduction in T stage after NAC was observed in 46.8 % of the patients with ILC and 65 % of the patients with IDC. Furthermore, a pCR was achieved for only 1 of 20 patients with ILC compared with 1 of 5 patients with IDC, which is comparable with previous literature.^17^ Downstaging was more frequently observed in the patients who presented with larger, hormone receptor-negative tumors in both histologic subtypes.

From previous studies, we know that patients with IDC are more likely to experience a pCR when their tumors are ER- and PR-negative and HER2-positive.^18^–^20^ In our cohort, these characteristics not only predicted a pCR but also were more frequently observed in tumors downstaged by NAC. Thus, tumor characteristics that predict who will respond to NAC also provide important information for further surgical treatment options (e.g., primary mastectomy or BCS).

Irrespective of the histologic tumor type, our study showed a remarkably low percentage of patients undergoing BCS after NAC (17.8 % in ILC and 37.5 % in IDC), resulting in a high rate of primary mastectomies in both histologic entities. These BCS rates are lower than we would expect from the data in recent literature. In a meta-analysis of Petrelli and Barni,^17^ which reviewed 17 studies on the response after NAC in ILC, pooled BCS rates of 35.4 % for ILC and 54.8 % for IDC were reported. Another study by Loibl et al.^20^ that pooled nine randomized trials calculated a BCS rate of 59.1 % in ILC and 71.1 % in IDC.

The patients in our cohort were derived from a national population-based registry and therefore are an objective representation of daily clinical practice in the Netherlands. We were unable to determine whether there were patients who were candidates for BCS but chose a mastectomy. Because mastectomy was performed for more than 50 % of the patients with cT1 disease, it appears that preferences of the patient, the physician, or both instead of tumor diameter alone influenced the surgical treatment plan, as mentioned in a previous study regarding BCS after NAC.^12^ The high mastectomy rate among ILC patients might be influenced by a certain degree of uneasiness and bias among surgeons regarding the treatment of ILC, provoking a decision to use “safe surgery”, with primary mastectomies as a result.^21^ Re-excision of lumpectomy margins after NAC was more often required for patients with lobular histology. Other studies have confirmed the significantly higher number of involved resection margins in ILC patients.^13^,^19^

Also, in the multivariate analysis, lobular histology was independently associated with the use of mastectomy. Evidence of the association between lobular histology and higher mastectomy rates after NAC is accumulating. This study confirmed the influence of histologic subtypes on treatment choices. To determine the effect of NAC on breast conservation rates in lobular histology, we compared patients who underwent NAC and those who had primary surgery. Our study showed that despite a significantly lower pCR among patients with ILC than among those who underwent surgery without NAC, patients with cT2 and cT3 breast cancer still can benefit from NAC regarding the chance of BCS, although the NNT remains high in this group. The NNT is 11.5 for cT2 and 15.4 for cT3 disease. To our knowledge, this effect has not been shown previously. A study by Boughey et al. 12 showed no benefit of NAC with regard to breast conservation in ILC patients. This study was the first to investigate the efficacy of NAC in terms of surgical outcome for ILC patients and to compare ILC patients after NAC with patients who underwent primary surgery. However, only a small number of patients were included in the study. Other studies concerning this subject were not stratified for timing of surgery (with or without NAC) or tumor size and are therefore not suitable for demonstrating an effect of NAC in subsets of patients with ILC.^11^,^22^,^23^

Due to the retrospective character of this study, some limitations need to be considered when the results are interpreted. Although our data were derived from a large nationwide database, some crucial information about specific tumor type such as grade and exact tumor diameter were not available. However, clinical and pathologic T-stages according to the TNM classification were extracted for each patient. Unfortunately, no information on the MRI use for our included patients was available to determine radiologic tumor response. Therefore, in this study, we compared clinical tumor stage at initial diagnosis with pathologic tumor stage to define downstaging in our cohort. Also, exact pathologic margin status was missing in 30 % of the cases. However, we did have the exact data on the number of surgical re-excisions in the setting of inadequate margins, which provided reliable information.

In conclusion, the findings show that BCS is less frequently achieved after NAC in patients with lobular histology compared with ductal histology. It appears that BCS can be achieved more frequently for patients with a cT2 or cT3 ILC when NAC is administered. However, the NNT is high in this group, illustrating the need to be modest about the clinical impact of NAC for patients with ILC. Future research should focus on determining reliable predictors of response to NAC among these patients to guide the selection for BCS.

## References

[1] Arpino G, Bardou VJ, Clark GM, Elledge RM (2004). Infiltrating lobular carcinoma of the breast: tumor characteristics and clinical outcome. Breast Cancer Res..

[2] Li CI, Uribe DJ, Daling JR (2005). Clinical characteristics of different histologic types of breast cancer. Br J Cancer..

[3] Katz A, Saad ED, Porter P (2007). Primary systemic chemotherapy of invasive lobular carcinoma of the breast. Lancet Oncol..

[4] Sastre-Garau X, Jouve M, Asselain B (1996). Infiltrating lobular carcinoma of the breast: clinicopathologic analysis of 975 cases with reference to data on conservative therapy and metastatic patterns. Cancer..

[5] Pestalozzi BC, Zahrieh D, Mallon E (2008). Distinct clinical and prognostic features of infiltrating lobular carcinoma of the breast: combined results of 15 international breast cancer study group clinical trials. J Clin Oncol..

[6] Waljee JF, Hu ES, Newman LA (2008). Predictors of re-excision among women undergoing breast-conserving surgery for cancer. Ann Surg Oncol..

[7] Fortunato L, Mascaro A, Poccia I (2012). Lobular breast cancer: same survival and local control compared with ductal cancer, but should both be treated the same way? Analysis of an institutional database over a 10-year period. Ann Surg Oncol..

[8] Van den Broek N, van der Sangen MJ, van de Poll-Franse LV (2007). Margin status and the risk of local recurrence after breast-conserving treatment of lobular breast cancer. Breast Cancer Res Treat..

[9] Vriens BE, Aarts MJ, de Vries B (2013). Doxorubicin/cyclophosphamide with concurrent versus sequential docetaxel as neoadjuvant treatment in patients with breast cancer. Eur J Cancer..

[10] Cristofanilli M, Gonzalez-Angulo A, Sneige N (2005). Invasive lobular carcinoma classic type: response to primary chemotherapy and survival outcomes. J Clin Oncol..

[11] Tubiana-Hulin M, Stevens D, Lasry S (2006). Response to neoadjuvant chemotherapy in lobular and ductal breast carcinomas: a retrospective study on 860 patients from one institution. Ann Oncol..

[12] Boughey JC, Wagner J, Garrett BJ (2009). Neoadjuvant chemotherapy in invasive lobular carcinoma may not improve rates of breast conservation. Ann Surg Oncol..

[13] Soucy G, Bélanger J, Leblanc G (2008). Surgical margins in breast-conservation operations for invasive carcinoma: does neoadjuvant chemotherapy have an impact?. J Am Coll Surg..

[14] Straver ME, Rutgers EJ, Rodenhuis S (2010). The relevance of breast cancer subtypes in the outcome of neoadjuvant chemotherapy. Ann Surg Oncol..

[15] Schouten LJ, Hoppener P, van den Brandt PA, Knottnerus JA, Jager JJ (1993). Completeness of cancer registration in Limburg, the Netherlands. Int J Epidemiol..

[16] Sobin LH (2002). International Union Against Cancer TNM Classification of Malignant Tumors.

[17] Petrelli F, Barni S (2013). Response to neoadjuvant chemotherapy in ductal compared to lobular carcinoma of the breast: a meta-analysis of published trials including 1764 lobular breast cancer. Breast Cancer Res Treat..

[18] Delpech Y, Coutant C, Hsu L (2013). Clinical benefit from neoadjuvant chemotherapy in oestrogen receptor-positive invasive ductal and lobular carcinomas. Br J Cancer..

[19] Fitzal F, Mittlboeck M, Steger G (2011). Neoadjuvant chemotherapy increases the rate of breast conservation in lobular-type breast cancer patients. Ann Surg Oncol..

[20] Loibl S, Volz C, Mau C (2014). Response and prognosis after neoadjuvant chemotherapy in 1051 patients with infiltrating lobular breast carcinoma. Breast Cancer Res Treat..

[21] Truin W, Roumen RM, Siesling S (2015). Patients with invasive lobular breast cancer are less likely to undergo breast-conserving surgery: a population-based study in the Netherlands. Ann Surg Oncol..

[22] Mathieu MC, Rouzier R, Llombart-Cussac A (2004). The poor responsiveness of infiltrating lobular breast carcinomas to neoadjuvant chemotherapy can be explained by their biological profile. Eur J Cancer..

[23] Cocquyt VF, Blondeel PN, Depypere HT (2003). Different responses to preoperative chemotherapy for invasive lobular and invasive ductal breast carcinoma. Eur J Surg Oncol..

